# Genome-Wide Identification and Chilling Stress Analysis of the NF-Y Gene Family in Melon

**DOI:** 10.3390/ijms24086934

**Published:** 2023-04-08

**Authors:** Meng Li, Qingjie Du, Juanqi Li, Hu Wang, Huaijuan Xiao, Jiqing Wang

**Affiliations:** College of Horticulture, Henan Agricultural University, Zhengzhou 450002, China

**Keywords:** *Cucumis melo*, nuclear factor Y, expression profiles, cold tolerance

## Abstract

The nuclear factor Y (NF-Y) transcription factor contains three subfamilies: NF-YA, NF-YB, and NF-YC. The NF-Y family have been reported to be key regulators in plant growth and stress responses. However, little attention has been given to these genes in melon (*Cucumis melo* L.). In this study, twenty-five *NF-Ys* were identified in the melon genome, including six *CmNF-YAs*, eleven *CmNF-YBs*, and eight *CmNF-YCs*. Their basic information (gene location, protein characteristics, and subcellular localization), conserved domains and motifs, and phylogeny and gene structure were subsequently analyzed. Results showed highly conserved motifs exist in each subfamily, which are distinct between subfamilies. Most *CmNF-Ys* were expressed in five tissues and exhibited distinct expression patterns. However, *CmNF-YA6*, *CmNF-YB1/B2/B3/B8*, and *CmNF-YC6* were not expressed and might be pseudogenes. Twelve *CmNF-Ys* were induced by cold stress, indicating the NF-Y family plays a key role in melon cold tolerance. Taken together, our findings provide a comprehensive understanding of *CmNF-Y* genes in the development and stress response of melon and provide genetic resources for solving the practical problems of melon production.

## 1. Introduction

Nuclear factor Y (NF-Y), also known as heme activator protein (HAP) or CCAAT binding factor, is widely distributed and evolutionarily conserved in eukaryotes such as yeast, mammals, and plants [1]. NF-Y is composed of three subunits: NF-YA (CBF-B/HAP2), NF-YB (CBF-A/HAP3), and NF-YC (CBF-C/HAP5). Generally, NF-YB and NF-YC recognize each other and interact through the HFM domain to form the NF-YB/C heterodimer in the cytoplasm. Then, the dimer will enter the nucleus, recognize with specific NF-YA and conduct secondary assembly to form a complete NF-Y heterotrimer complex (NF-YA/B/C) [2] or form a heterotrimer with another factor (X) in the nucleus (NF-YB/C-X) [3]. The trimer complex can regulate target genes through the N-terminal of the NF-YA subunit or X factor, the C-terminal transcriptional activation domain of the NF-YC subunit (glutamine rich hydrophobic domain), or recruit other transcription factors to regulate target genes through protein interaction [4,5].

Emerging research has proved that *NF-Ys* are widely involved in plant growth and development, including embryo morphogenesis, seed germination, hypocotyl and root elongation, flowering regulation, and fruit ripening [6,7]. AtNF-YC3/C4/C9 has been shown to be involved in regulating gibberellin and ABA mediated seed germination in *Arabidopsis* [8]. OsNF-YB1 can directly target sucrose transporter genes to affect the rice grain filling [9]. AtNF-YA2/10 and PdNF-YB21 promote taproot growth by regulating cell division and elongation [10,11]. Intriguingly, NF-Y subunit participates in the regulation of flowering time in a complex mechanism, mainly through the gibberellin pathway [12], ageing [13], photoperiod [14], and the stress pathway [15]. In addition, it was found that three *SlNF-YAs* and two *SlNF-YBs* affected the ripening of tomato fruit [16]. In banana, MaNF-YA5/B1/B2/C9/C11/C14 and MaNF-YA1/A3/A6/B3/B6/C2/C5, as transcriptional activators and inhibitors, respectively, participate in postharvest fruit maturation [17]. In addition to participating in plant growth and development, the NF-Y family also plays an important role in coping with abiotic stresses [1].

AtNF-YC1 confers *Arabidopsis* cold tolerance by directly targeting the CCAAT box in the *AtXTH21* (xyloglucan endotransglucosylase/hydrolase 21) promoter [18]. AtNF-YC10, AtNF-YA2, and AtNF-YB3 form a heterotrimer complex, which combines with *DREB2A* (dehydration-responsive element binding protein 2) under heat stress [19]. Moreover, *AtNF-YB2* [19], *OsNF-YA7* [20], *ZmNF-YB16* [21], and *PdNF-YB21* [10] all contribute to plant drought tolerance. *AtNF-YA1* [22], *OsNF-YC13* [23], and *HvNF-YA1/A6* [24] are associated with plant salt tolerance. Recently, with the publication of the genome, a large number of *NF-Y* genes were found to be induced by cold, heat, salt, drought, or other stresses in various plants, such as grape [25], tea plant [26], petunia [27], cucumber [6], and poplar [28]. Although extensive studies of the *NF-Y* gene family have been identified and functionally annotated in other plant species, little is known in melon.

Melon (*Cucumis melo* L.) is an economical crop that is widely cultivated worldwide. Low temperature [29] was one of the main limiting factors affecting melon production. Since the melon genomic data was released [30], numerous TF families associated with melon plant growth and stress response have been identified [31,32,33,34,35]. In analyzing the cold tolerance mechanism of melon, we found that some *CmNF-Ys* were involved based on transcriptome sequencing, but the *NF-Y* family in melon has not been characterized yet. To explore the evolutionary relationship, gene and protein structure, possible trimer forms, and potential roles of melon *NF-Y* members, especially in cold tolerance, we performed a genome-wide identification of the family members. A total of 25 *CmNF-Ys* (6 *NF-YAs*, 11 *NF-YBs*, and 8 *NF-YCs*) were identified in the melon genome. We adopted bioinformatics to analyze their features and potential functions based on publicly available data or experiments and focused on the expression patterns of *CmNF-Ys* in response to low temperature. Furthermore, we also collected the expression profiles of *CmNF-Ys* in different tissues and different fruit development stages. Taken together, our results provide a set of candidate *CmNF-Y* genes for future study and genetic modification in melon.

## 2. Results

### 2.1. Identification and Characterization of Melon NF-Y Genes

After searching, screening and BLAST analysis, a total of 25 *CmNF-Y* genes were identified, including 6 *CmNF-YA*, 11 *CmNF-YB*, and 8 *CmNF-YC* genes (Table 1). They were unevenly distributed on 10 chromosomes. We renamed the *CmNF-Y* genes according to their subfamily branch and chromosome locations, renaming them from *CmNF-YA1* to *CmNF-YA6*, *CmNF-YB1* to *CmNF-YB11,* and *CmNF-YC1* to *CmNF-YC8*. For the three CmNF-Y subfamilies, the protein lengths of CmNF-YAs ranged from 169 aa (amino acid) to 341 aa, showing the longest average length (280.2 aa), while those of CmNF-YBs ranged from 117 aa to 225 aa with the shortest average length (170.4 aa), and of CmNF-YCs ranged from 117 aa to 282 aa with medium average length (230.3 aa). The predicted MW of the 25 CmNF-Ys ranged from 12.89 kDa (CmNF-YC6) to 37.07 kDa (CmNF-YA4), and the predicted pI ranged from 4.93 (CmNF-YC5) to 9.52 (CmNF-YC8). The instability index ranged from 25.58% (CmNF-YB3) to 71.76% (CmNF-YC1). Only CmNF-YB2, CmNF-YB3, and CmNF-YB11 were stable proteins with the instability index less than 40%, and the others were unstable. The aliphatic index ranged from 48.35 (CmNF-YB11) to 87.61 (CmNF-YC6). Except for CmNF-YC6 (GRAVY = 0.035), all GRAVY were negative (GRAVY < 0), which belongs to hydrophilic protein. The subcellular localization of *CmNF-Ys* was predicted by Euk-mPLoc 2.0. Results showed that 23 members were located in the nucleus, and only *CmNF-YC2* and *CmNF-YC3* were located in both the nucleus and cytoplasm (Table 1).

### 2.2. Gene Structure of CmNF-Ys

**Figure 1:**
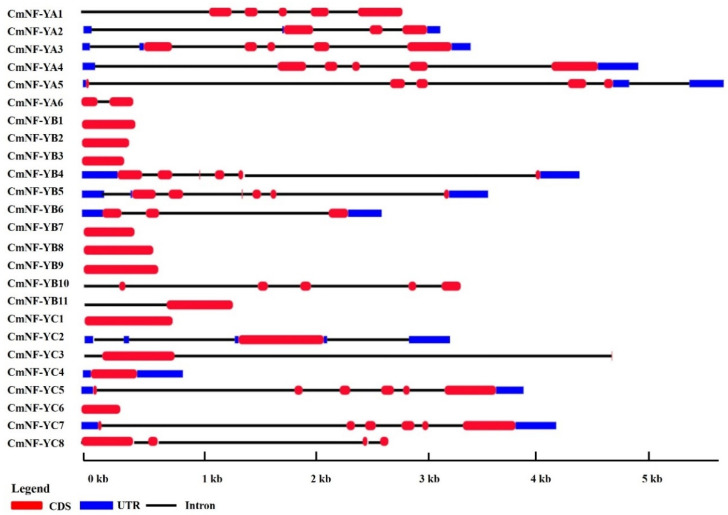
Gene structure of *CmNF-Y* genes. The red boxes and black lines indicate exons and introns, respectively. The blue boxes indicate untranslated regions. Their lengths can be estimated by the scale bar at the bottom.

The gene structure of *CmNF-YAs*, *CmNF-YBs,* and *CmNF-YCs* varied greatly (Figure 1). Overall, the intron numbers varied from 0 to 6, with nine members having no introns and 14 members having no untranslated region (UTR) (Figure 1). Most *CmNF-YAs* contained five introns, with the exception of *CmNF-YA2* and *CmNF-YA6*, which had three and one introns, respectively. Among the *CmNF-YBs*, *CmNF-YB5* contained six introns, *CmNF-YB4*/*B10* contained five introns; *CmNF-YB6* and *CmNF-YB11* had two and one introns, respectively (Figure 1), while the six other *CmNF-YBs* were intron-less (Figure 1). As for the *CmNF-YCs*, *CmNF-YC5* and *CmNF-YC7* both harbored five introns; *CmNF-YC2*, *CmNF-YC8* and *CmNF-YC3* contained three, three, and two introns, respectively, while *CmNF-YC1*/*C4*/*C6* had no introns (Figure 1).

### 2.3. Motif Composition and Multiple Alignments of the CmNF-Ys

**Figure 2:**
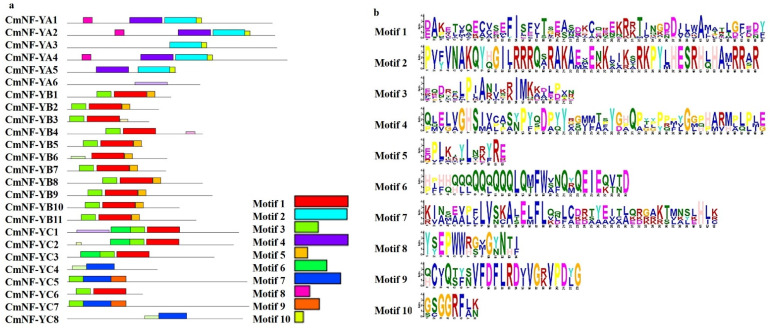
Conserved motifs prediction of CmNF-Y proteins using the MEME program. (**a**) Distribution of CmNF-Y conserved motifs. The ten motifs were displayed in different colored boxes. (**b**) The sequence logos of ten conserved motifs.

**Figure 3:**
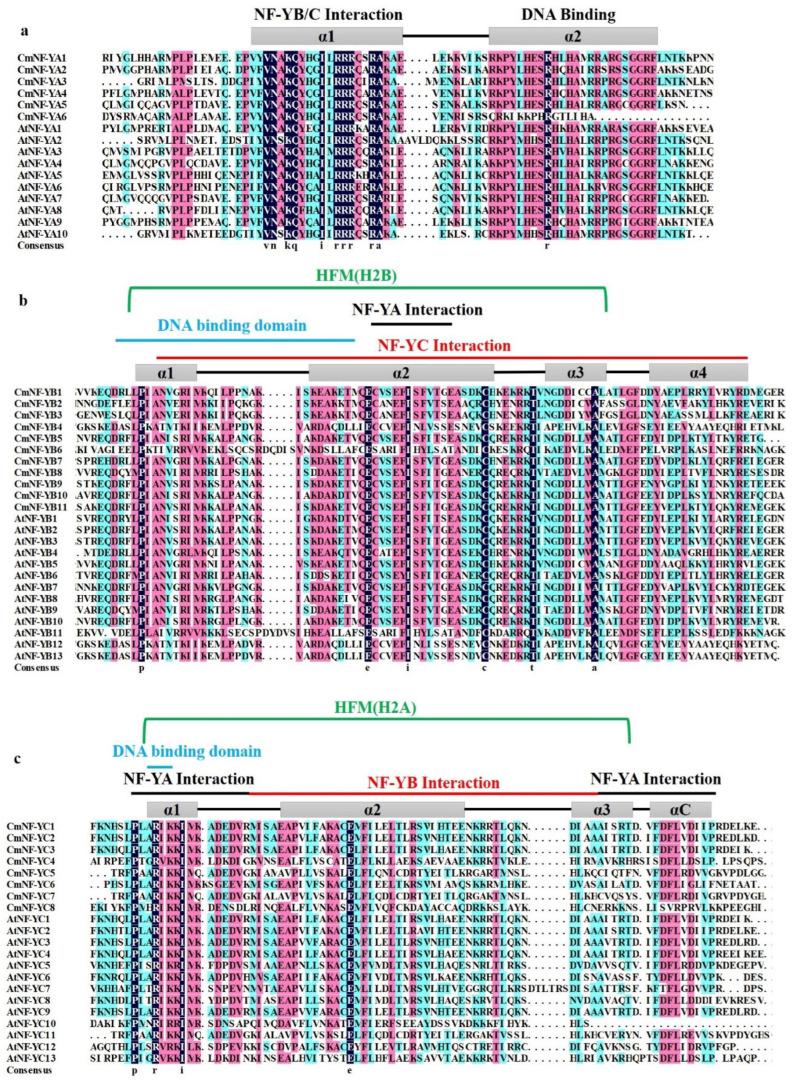
Alignments of CmNF-Y and AtNF-Y domains. The sequences of melon NF-YAs (**a**), NF-YBs (**b**), and NF-YCs (**c**) were aligned with corresponding referred sequences from *Arabidopsis thaliana*. The different colors represent the level of sequence homology, with black ≥ 100%, purple ≥ 75% and blue-green ≥ 50%.

Combining MEME motif (Figure 2) and multiple alignments (Figure 3), the results showed that there were significant differences in conserved regions among the three subfamilies. The CmNF-YA conserved domains consisted of two α-helices, while the CsNF-YB and CmNF-YC were composed of four α-helices (Figure 3). CmNF-YAs were more conservative, mainly containing motif 2 (light blue), motif 4 (purple), and motif 10 (fluorescent green) excluding CmNF-YA6 (Figure 2). These three motifs were the two core subdomains of NF-YA, one responsible for the NF-YB/C interaction and the other for DNA binding (Figure 3a) [36]. CmNF-YBs and CmNF-YCs harbored the HFM of the H2B and H2A, respectively, which were conserved regions for DNA binding and subgroup member interactions (Figure 3b,c) [36]. However, CmNF-YBs were the most conserved, and their conserved motifs were uniform, including motif 1 (red), motif 3 (light green), and motif 5 (orange) (Figure 2), while CmNF-YCs were the most diverse, motif1, motif 3, motif 6, motif 7, and motif 9 were unevenly distributed among the eight members, implying the diversity of their functions.

### 2.4. Secondary and Tertiary Structure Prediction of CmNF-Ys

The secondary structure of CmNF-Y proteins was predicted by PRABI software. Results showed that the secondary structure was composed of α-helix, extended strand, β-turn, and random coil. Among them, α-helix and random coil were the main components, followed by extended strand, and β-turn accounted for the smallest proportion (Table 2). For CmNF-YAs, random coil accounted for the greatest proportion, ranging from 49.70% (CmNF-YA5) to 70.97% (CmNF-YA4). For CmNF-YBs, α-helix accounted for more than 40% (except CmNF-YB9, 39.56%) and up to 65.16% (CmNF-YB6). The random coil of most CmNF-YBs accounts for 30% to 45% and up to 53.77% (CmNF-YB9). For CmNF-YCs, the proportion of α-helix and random coil was almost equal, about 30% to 55%.

**Figure 4:**
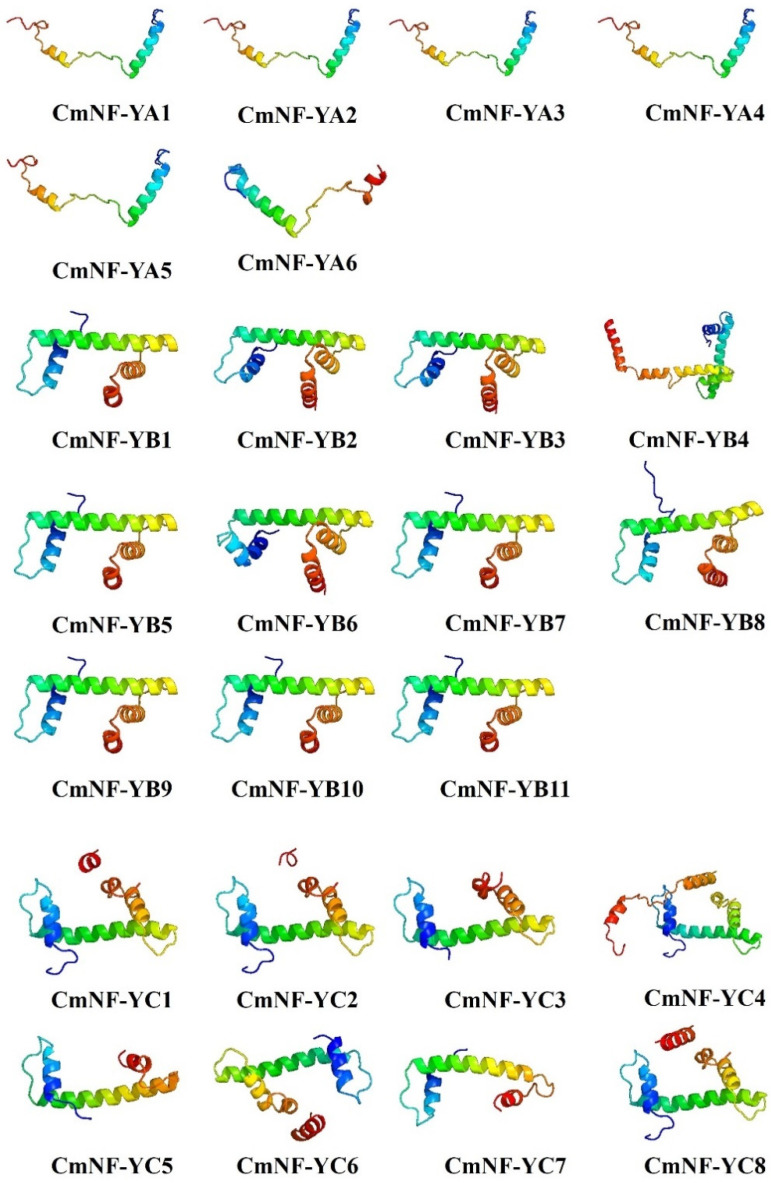
Tertiary structure of CmNF-Ys predicted by homologous modeling.

To further compare the protein tertiary structures among the CmNF-Y members, protein 3D models were constructed by homologous modeling (Figure 4). The 3D models of CmNF-YA, CmNF-YB, and CmNF-YC member proteins were based on template c6r2vA, c7c9Ob, and c7cvqF, respectively. There were two α-helices in the tertiary structure of CmNF-YAs, and four α-helices in both CmNF-YBs and CmNF-YCs. The prediction models of many members of CmNF-YAs and CmNF-YBs were the same, such as CmNF-YA1/A2/A3/A4/A5, CmNF-YB2/B3, and CmNF-YB1/B5/B7/B9/B10/B11, proving the conservatism of their functions. However, the spatial structure of CmNF-YCs was quite different, suggesting the diversity of its functions.

### 2.5. Phylogenetic Analysis of CmNF-Ys

**Figure 5:**
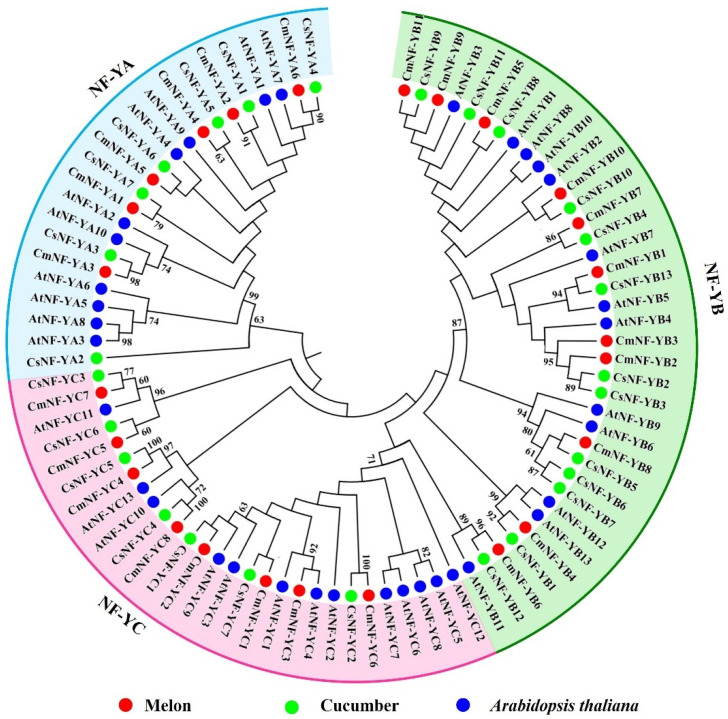
Molecular phylogenetic analysis of 88 NF-Y proteins from melon (*Cucumis melo* L.), cucumber (*Cucumis sativus*), and *Arabidopsis thaliana*. MEGA 11 was used to build the maximum likelihood (ML) tree with 1000 bootstrap replicates.

The NF-Y family members of three plants, *Arabidopsis thaliana* (36), cucumber (27), and melon (25), were used to construct a maximum likelihood (ML) phylogenetic tree with MEGA11.0 (Figure 5). All 88 members were automatically grouped into three clusters: NF-YAs, NF-YBs, and NF-YCs (Figure 5). On the whole, the phylogeny between melon and cucumber was higher than that of *Arabidopsis*. Almost all CmNF-Ys have one-to-one corresponding members in cucumber (Figure 5), and their sequence homology ranges from 81.1% to 100%, with an average of 94.4% (Table S1). Only CmNF-YC3 clustered with AtNF-YC1 and AtNF-YC4 (Figure 5), and the sequence similarity was 86.3% and 82.4%, respectively (Table S1).

### 2.6. Protein–Protein Association Network Analysis of CmNF-Ys

**Figure 6:**
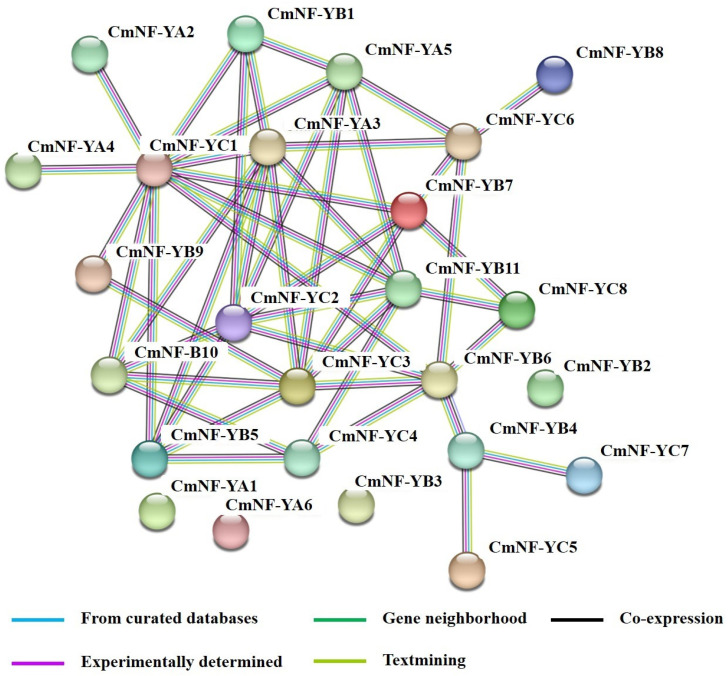
Protein–protein association network analysis of CmNF-Ys.

In theory, there were 528 (6 × 11 × 8) heterotrimeric complexes among the 25 members of melon. When predicted with the STRING database, there were still 71 heterotrimeric complexes (NF-YA+B+C) when the confidence level was set to the highest value (0.900) (Figure 6). Among them, CmNF-YA3/A5, CmNF-YB5/B6/B7/B10/B11, and CmNF-YC1/C2/C3/C6 occupy important positions in the interacting network and crosstalk with at least 5 or more members, among which CmNF-YC1 has reciprocal interactions with 11 members (Figure 6).

### 2.7. Expression Profiles of CmNF-Ys in Various Tissues and Fruits during Development

**Figure 7:**
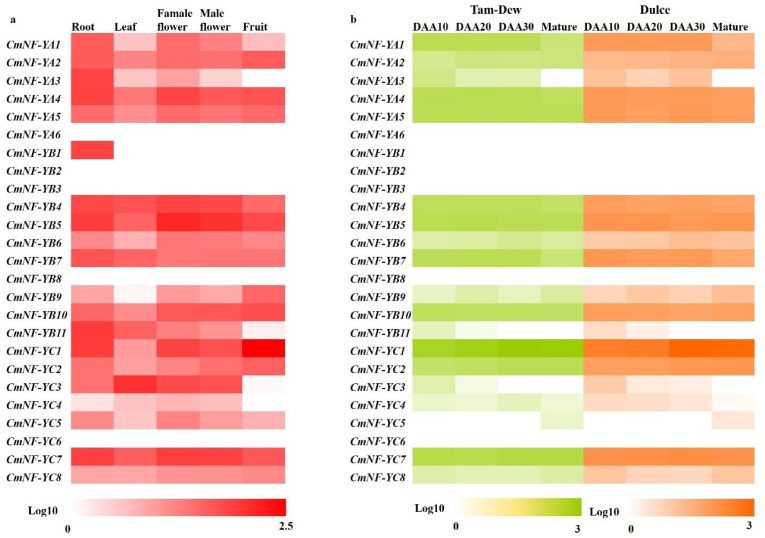
Expression profiles of *CmNF-Ys* in five melon tissues (**a**) and different fruit development stages (**b**) based on the transcriptome data. The RPKM values were transformed to log10. The expression in various melon tissues was shown, including root, leaf, female flower, male flower, and fruit. Tam-Dew is a green flesh, non-climacteric line. Dulce is an orange flesh, climacteric line. DAA: days after anthesis.

The transcript levels of 25 *CmNY-Fs* in different tissues and different fruit development stages were searched in 2 transcriptomes (PRJNA383830 and PRJNA286120). The data was employed to construct heatmaps (Figure 7). As shown in Figure 7, there were five genes, *CmNF-YA6*, *CmNF-YB2*/*B3*/*B8* and *CmNF-YC6*, almost unexpressed in the five tissues (RPKM < 1). *CmNF-YA3*, *CmNF-YB11*, *CmNF-YC3,* and *CmNF-YC4* were only not expressed in fruit (Figure 7a). Interestingly, *CmNF-YB1* was only expressed in roots (Figure 7a). In addition, other genes were expressed in roots, leaves, female flowers, and male flowers, among which *CmNF-YB11*, *CmNF-YC3*, *CmNF-YB5,* and *CmNF-YC1* were most expressed in roots, leaves, flowers (female and male flowers), and fruit, respectively (Figure 7a).

Dulce and Tam-Dew are climacteric and non-climacteric lines, respectively [37]. During fruit development, the expression of *CmNF-Ys* in two genotypes was basically the same, and nearly half of the genes were constantly and steadily expressed (Figure 7b). There were six *CmNF-Ys* (*CmNF-YA6/B1/B2/B3/B8/C6*) that were undetected during the whole process of fruit development (RPKM<1) (Figure 7b). Notably, *CmNF-YC1* maintained the highest expression in the four stages and increased with fruit development and maturity; on the contrary, the expression of *CmNF-YA3*, *CmNF-YB11,* and *CmNF-YC3* decreased continuously (Figure 7b). Intriguingly, the expression of *CmNF-YA1* and *CmNF-YB7* was basically stable within 30 DAA, and only decreased sharply at maturity, suggesting that they might be involved in the regulation of fruit ripening (Figure 7b). These results indicated that the six genes might regulate melon fruit ripening, among which *CmNF-YC1* might be the principal gene.

### 2.8. Expression Profiles of CmNF-Ys under Cold Stress

**Figure 8:**
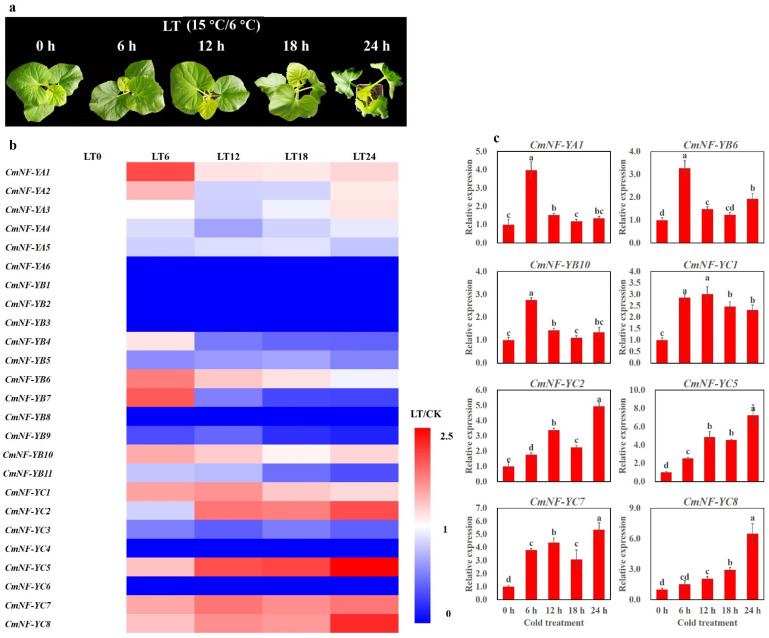
Expression profiles of *CmNF-Ys* under cold stress. The seedlings were exposed to cold stress (15 °C/6 °C, day/night, LT) for 24 h. The third true leaves were sampled for RNA-Seq and qRT-PCR after 0, 6, 12, 18, and 24 h cold treatment. We calculated the relative value before and after cold treatment (LT/CK) and converted the data into log_2_ to draw the heat map. Error bars indicate the standard error of the mean. A one-way ANOVA was performed using Duncan’s multiple-range test. Different lowercase letters represent significant differences at the 0.05 level.

As shown in Figure 8a, low temperature caused melon to lose water and wilt gradually. To obtain candidate genes for cold tolerance in melon, we did RNA-seq and qRT-PCR analysis in melon seedling stage. There were 13 *CmNF-Ys* that were severely inhibited or not expressed under low temperature (blue areas). On the contrary, 12 *CmNF-Y*s were up-regulated (Figure 8b). Among them, *CmNF-YA2/A3* and *CmNF-YB4/B7* were up-regulated only at one or two stages during cold treatment (Figure 8b). While other genes, such as *CmNF-YA1*, *CmNF-YB6/B10,* and *CmNF-Y*C*1/C2/C5/C7/C8*, were up-regulated throughout almost the whole process (Figure 8b). These eight genes were selected for qRT-PCR analysis of gene expression under cold stress. The results were generally consistent with the changes of transcriptome data (Figure 8c). These results showed that *CmNF-Ys* might play an important role in melon response to cold stress.

## 3. Discussion

The NF-Y family is widely distributed in eukaryotic organisms, including yeast, plants, and mammals. In mammals and yeast, all three NF-Y subunits are encoded by a single gene [1]. However, *NF-Y* genes undergo replication and expansion in plants, and each subunit is encoded by a gene family. This provides more NF-YA/NF-YB/NF-YC trimer combinations, increasing the complexity of NF-Y function [3]. NF-Y transcription factors recognize and bind the CCAAT box, which exists in the promoter region of 20~30% of eukaryotic genes [36]. It is proven that the NF-Y family is widely involved in plant growth, fruit maturation, and stress response. To date, the NF-Y family has been identified in a variety of crops [7,17,26,28,38,39]. However, there are few reports about the melon NF-Y family. How NF-Y members participate in the growth, development, and stress response in melon is still unknown.

In this study, a total of 25 *CmNF-Y* genes were identified (Table 1), which was similar to the number in other cucurbits, such as cucumber [6] and watermelon [38]. However, this is obviously less than that of other crops, such as the 36 in Arabidopsis [5], 59 in tomato [16], and 60 in alfalfa [39], indicating that the NF-Y genes amplification degree of cucurbits is relatively small. Alternatively, there may also be replication gene loss events in the evolution process [40,41]. Each member of the subgroup had the characteristic structure, conservative domain, and motif composition of the corresponding subunit (Table 1, Figure 2, Figure 3 and Figure 4), which proved that melon NF-Ys were relatively conservative in evolution. However, *NF-YA6*, *NF-YB1/B2/B3/B8,* and *NF-YC6* were almost not expressed in the tissues (roots, leaves, female flowers, male flowers, and fruits) (RPKM < 1) (Figure 7a) and have almost no effect on fruit development (Figure 7b) and cold tolerance (Figure 8). The commonness of these genes is that they differ greatly from the corresponding subgroups in structure, showing the lack of UTR regions and few or no introns (Figure 1). That is, there is a defect in the 5’-terminal promoter region, or there is a lack of intron sequence compared with the corresponding normal gene, which is in accord with the characteristics of processed pseudogenes [42]. Therefore, it is speculated that these genes might be pseudogenes. On the contrary, other genes might play multiple roles in different ways, in melon. NF-Y factors usually form heterotrimeric complexes to perform functions, mainly by directly binding to CCAAT cis-acting elements in the promoter region of target genes in the nucleus, and then regulating them or interacting with other factors to jointly activate or inhibit the expression of target genes [4,43]. The 25 CmNF-Ys in melon could form 528 trimer complexes, in theory. Even if the prediction threshold was set to the highest value, there were still 71 heterotrimers (Figure 6), but the specific interaction results need to be verified by a yeast two-hybrid or three-hybrid test (Myers and Holt, 2018).

Increasing evidence has shown that NF-Y is involved in regulating the flowering of plants, such as *AtNFY-B2/B3/C2/C3/C4/C9* [44,45], *PtNF-YB1* [46], and *ZmNF-YA3/C2* [47]. Intriguingly, most of the genes were highly expressed in melon female and male flowers, or reached the peak value, such as *CmNF-YA4/A5*, *CmNF-YB4/B5/B6/B10,* and *CmNF-YC1/C2/C3/C7/C8* (Figure 7a), implying that these genes might regulate the flowering of melon. Furthermore, *CmNF-YC1* was continuously up-regulated with maturity, which might be a transcriptional activator regulating maturation. On the contrary, *CmNF-YA1/A3*, *CmNF-YB7/B11,* and *CmNF-YC3* were negatively correlated with maturity and might act as transcriptional inhibitors. This is similar to the results on banana, there are six (*MaNF-YA5 /B1/B2/C9/C11/C14*) and seven (*MaNF-YA1/A3/A6/B3/B6/C2/C5*) genes in banana which are involved in positive regulation and negative regulation of ripening, respectively [17]. In addition, during the development of melon fruit, the expression of *CmNF-Ys* in climacteric fruit and non-climacteric fruit were almost the same (Figure 7b) indicating that the regulation of melon fruit development and maturation by *CmNF-Ys* might not be induced by ethylene.

The role of *NF-Ys* under abiotic and biotic stress gradually emerged, such as the involvement of *ZmNF-YB2/16* [21], *OsNF-YA4/A7* [20], and *PdNF-YB21* [10] in drought tolerance. *AtNFY-C1* (AtHAP5A) positively regulates cold tolerance [18], and *BnaMI169N-BnaNF-YA9* modulates responses to salt stress, drought, and ABA [48]. In melon, *CmNF-YA1*, *CmNF-YB6/10,* and *CmNF-YC1/C2/C5/C7/C8* were continuously up-regulated under cold stress. On the whole, *CmNF-YC* members were more involved (Figure 8), which was different from that in *Petunia hybrida*. *PhNF-YAs* were mainly involved in cold tolerance response [27]. In addition, *CmNF-YB4/B7* was only up-regulated after 6 h of cold treatment, which might play a role in the early cold response. Above all, the aforementioned genes might be candidate genes for cold tolerance in melon. A few studies have reported NF-Ys are involved in defending against pathogen infection [49,50,51]. For melon, powdery mildew (PM) and fusarium wilt (FOM) are the main limiting factors affecting melon production [52,53]. Preliminary analysis showed that *CmNF-YB7/C3* (Figure S1) and *CmNF-YA4/A5/B5/C2/C7* (Figure S2) played a role in defensing fusarium wilt and PM, respectively, implying their possible involvement in melon immune signaling. In conclusion, here, we provide an overview of melon *NF-Y* genes and a reference for further study on the functions of *NF-Y* in melon under biotic and abiotic stresses. This study is only the beginning of the research on NF-Ys in melon, and the functions of many candidate genes have not been verified yet. In the future, we will further elucidate the gene functions of these candidate genes in biotic and abiotic stresses, especially *CmNF-YC1*. Yeast two-hybrid or three-hybrid assays can be used to screen for reciprocal NF-YA, NF-YB, or other transcription factors and to deeply elucidate the molecular mechanisms involved in melon cold tolerance. In addition, the role of NF-Ys in melon growth, development, and fruit ripening can also be further investigated.

## 4. Materials and Methods

### 4.1. Plant Materials and Cold Treatment

Melon ‘L5283’ were used herein in accordance with our previous study [54]. Seedlings were cultured in a growth chamber by using the commercial medium (peat:vermiculite = 3:1). At the one-leaf stage, seedlings were transplanted into plastic pots (7 cm × 7 cm). The seedlings were grown under 28 °C/18 °C (day/night) and 60% relative humidity. At the three-leaf stage, the seedlings were exposed to cold stress (15 °C/6 °C, day/night) for 24 h. This temperature simulated the cold stress in winter and spring according to the actual practice [55]. The third true leaves (from the bottom) were sampled after 0, 6, 12, 18, and 24 h cold treatment. All samples were immediately frozen in liquid nitrogen and kept at −80 °C for total RNA extraction. At least three biological repeats were performed for each treatment.

### 4.2. Identification of NF-Y Family Members in Melon

Firstly, the key words ‘nuclear factor Y’ were searched in the melon genome database [Melon (DHL92) v3.6.1 Genome, http://cucurbitgenomics.org/organism/, accessed on 12 December 2022] [18]. Meanwhile, the amino acid sequences of all 36 *Arabidopsis* NF-Ys were downloaded from the NCBI website (https://www.ncbi.nlm.nih.gov/, accessed on 12 December 2022) for protein BLAST in melon database, the expected threshold of e-Value was set to 1e^−2^. The candidate *NF-Y* members were obtained by merging the above two methods. Candidate *NF-Y* genes were confirmed if they contained at least one NF-Y domain (PF02045 and PF00808) according to the Hidden Markov Model (HMM) profile in the Pfam database (http://pfam.janelia.org/, accessed on 12 December 2022) and SMARAT (http://smart.embl-heidelberg.de/, accessed on 12 December 2022).

### 4.3. Analysis of Basic Characteristics of CmNF-Y Members

The position of *CmNF-Ys* on the chromosome, coding sequence, and amino acid sequence were obtained from Melon (DHL92) v3.6.1 Genome. The physicochemical properties of CmNF-Y proteins, including molecular weight (MW), theoretical isoelectric point (pI), instability index, aliphatic index, and hydrophilicity (GRAVY), were analyzed through the ExPASy-ProtParam tool (https://web.expasy.org/protparam/, accessed on 18 December 2022) [56]. Subcellular location was predicted by Euk-mPLoc 2.0 (http://www.csbio.sjtu.edu.cn/bioinf/euk-multi-2/, accessed on 18 December 2022) [57].

### 4.4. Multiple Sequence Alignment and Phylogenetic Analysis

For phylogenetic analysis, the protein sequences of NF-Ys from *Arabidopsis*, cucumber, and melon were used to generate a phylogenetic tree by MEGA 11 software (version 11.0.10). Parameter Settings were as follows: alignment: by muscle; phylogeny: maximum likelihood (ML) tree; test of phylogeny: bootstrap method, 1000 replications; and model: Poisson model. The EVOLVIEW online website (http://www.evolgenius.info/evolview, accessed on 25 December 2022) was used to beautify the evolutionary tree. Multiple sequence alignment of CmNF-Y and AtNF-Y conserved domain sequences by DNAMAN 7.0 software.

### 4.5. Genetic Structure and Conserved Motif Analyses

CmNF-Y protein sequences were submitted to the MEME Suite 5.4.1 to analyze the conserved motifs (https://meme-suite.org/meme/tools/meme, accessed on 1 January 2023). The parameters were set to classic mode, any number of repetitions (any), up to ten motifs, and others were set to default [58]. The gene structures of the *CmNF-Ys* were analyzed with the GSDS 2.0 program (http://gsds.cbi.pku.edu.cn/, accessed on 1 January 2023) [59].

### 4.6. Protein Secondary, Tertiary Structure, and Association Network Analysis

The protein secondary structure was predicted by PrabiSOPMA (https://npsa-prabi.ibcp.fr/cgi-bin/npsa_automat.plpage=npsa_sopma.html, accessed on 15 January 2023) and the tertiary structure was predicted by Protein Homology/analog Y Recognition Engine v 2.0 (Phyre^2^) (http://www.sbg.bio.ic.ac.uk/phyre2/html/page.cgi?id=index, accessed on 15 January 2023) [60]. The protein association network was analyzed by STRING v 11.5 (https://cn.string-db.org, accessed on 15 January 2023) [61].

### 4.7. RNA Isolation and Analysis of CmNF-Ys Expression Patterns

Total RNA was extracted with an ultrapure RNA Kit (Tiangen, Beijing, China) and template cDNA synthesis was performed using the PrimeScript™ RT Master Mix (Tiangen, Beijing, China) following the manufacturer’s instructions. A part of the template cDNA was used for RNA-Seq. Meanwhile, the template cDNAs amplified with a 20 μL of reaction solution by using TOROGreen^®^ qPCR Master Mix (Toroivd, Shanghai, China). The qRT-PCR program consisted of a preliminary step of 60 s at 95 °C, followed by 40 cycles at 95 °C for 10 s, and 60 °C for 30 s. The *Actin7* from melon was used as an internal control [55]. The relative gene expression level was calculated via the 2^−ΔΔCt^ method. Three biological and three technical replicates were used for each sample. All the primers used in this study are listed in Table S2. In addition, the raw transcriptome sequencing data was used to analyze the tissue specificity of NF-Y members and their roles in fruit ripening, including five melon plant tissues, including roots, leaves, male flowers, female flowers, and fruits (PRJNA383830) [62], as well as fruit development of green-fleshed (Dulce) and orange-fleshed (Tam-Dew) at 10, 20, and 30 days after anthesis (DAA) and maturity, respectively (PRJNA286120) [37].

### 4.8. Statistical Analysis

Data analyses were performed with Microsoft Excel 2019 and SPSS 20.0 software (SPSS, Chicago, IL, USA). An ANOVA was performed using Duncan’s multiple-range test at a level of *p* < 0.05.

## 5. Conclusions

**Figure 9:**
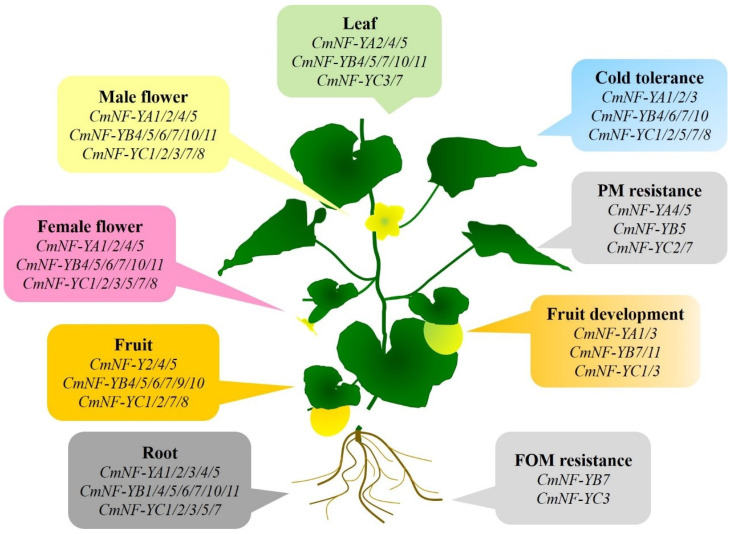
Schematic diagram of the probable function of melon *CmNF-Ys*. According to the tissue expression patterns and RNA-Seq data under stress, the probable biological processes that melon *CmNF-Ys* are involved in are summarized.

In this study, a total of 25 *CmNF-Ys* were identified, including six *CmNF-YAs*, eleven *CmNF-YBs*, and eight *CmNF-YCs*. Specific information on the protein characteristics/architecture, genomic structures, conserved domains/motifs, and phylogenetic analysis were presented. The members of each subgroup were conservative in evolution, among which *CmNF-YA6*, *CmNF-YB1/B2/B3/B8,* and *CmNF-YC6* might be pseudogenes. Other genes played an important role in melon growth and development, fruit ripening, and low temperature (Figure 9). Our results provide a basis for further studies on the functions of genes in the *CmNF-Y* family.

## Figures and Tables

**Table 1:** Identification of the *CmNF-Ys* gene family in melon.

Gene	Gene ID	Gene Locus	Protein						
			Size (aa)	MW (kDa)	pI	Instability Index (%)	Aliphatic Index	GRAVY	Subcellular Localization
*CmNF-YA1*	MELO3C023554	Chr01: 33657884 … 33661524 (−)	318	35.64	7.90	42.85	66.86	−0.784	Nucleus
*CmNF-YA2*	MELO3C015196	Chr02: 6195415 .. 6200409 (−)	322	35.43	7.15	57.36	54.84	−0.792	Nucleus
*CmNF-YA3*	MELO3C009551	Chr04: 31271731 .. 31275259 (+)	325	35.70	9.40	57.01	71.14	−0.473	Nucleus
*CmNF-YA4*	MELO3C014590	Chr05: 1114809 .. 1119924 (+)	341	37.07	6.53	54.09	49.62	−0.926	Nucleus
*CmNF-YA5*	MELO3C007077	Chr08: 594883 .. 611363 (+)	169	18.95	9.32	66.59	57.16	−0.193	Nucleus
*CmNF-YA6*	MELO3C023161	Chr08: 16875004 .. 16880763 (−)	206	23.47	8.94	68.50	55.44	−0.94	Nucleus
*CmNF-YB1*	MELO3C016042	Chr01: 32765510 .. 32766166 (−)	161	17.49	5.16	41.97	55.09	−0.816	Nucleus
*CmNF-YB2*	MELO3C015318	Chr02: 737865 .. 738293 (+)	142	16.50	5.17	39.28	65.99	−0.962	Nucleus
*CmNF-YB3*	MELO3C015320	Chr02: 743726 .. 744109 (+)	127	14.69	6.15	25.58	70.79	−0.754	Nucleus
*CmNF-YB4*	MELO3C017276	Chr02: 24713746 .. 24717503 (−)	210	23.58	5.05	65.10	74.81	−0.391	Nucleus
*CmNF-YB5*	MELO3C009309	Chr04: 32949453 .. 32953167 (−)	117	12.97	5.86	51.56	66.84	−0.669	Nucleus
*CmNF-YB6*	MELO3C014503	Chr05: 1870042 .. 1872775 (+)	155	17.48	5.01	48.06	79.29	−0.842	Nucleus
*CmNF-YB7*	MELO3C014103	Chr06: 36946833 .. 36947294 (+)	153	16.42	5.23	41.61	60.07	−0.614	Nucleus
*CmNF-YB8*	MELO3C014120	Chr06: 37108875 .. 37109569 (+)	210	22.86	5.58	45.97	66.90	−0.535	Nucleus
*CmNF-YB9*	MELO3C017568	Chr07: 25210242 .. 25211051 (−)	225	24.65	7.76	50.93	64.62	−0.633	Nucleus
*CmNF-YB10*	MELO3C011726	Chr10: 5430171 .. 5434588 (−)	174	19.00	5.11	44.08	64.02	−0.638	Nucleus
*CmNF-YB11*	MELO3C025951	Chr11: 14106203 .. 14107884 (+)	200	20.56	6.31	31.98	48.35	−0.648	Nucleus
*CmNF-YC1*	MELO3C018689	Chr01: 2153793 .. 2156247 (−)	266	29.96	5.96	71.76	63.53	−0.705	Nucleus
*CmNF-YC2*	MELO3C015332	Chr02: 804011 .. 809860 (−)	258	28.61	5.89	59.94	70.35	−0.517	Nucleus/Cytoplasm
*CmNF-YC3*	MELO3C026204	Chr02: 26758439 .. 26761732 (−)	228	24.99	5.07	65.10	71.58	−0.433	Nucleus/Cytoplasm
*CmNF-YC4*	MELO3C011458	Chr03: 25878114 .. 25879023 (+)	140	15.71	9.04	51.67	81.57	−0.57	Nucleus
*CmNF-YC5*	MELO3C014530	Chr05: 1604408 .. 1608430 (−)	279	31.13	4.93	42.67	71.29	−0.846	Nucleus
*CmNF-YC6*	MELO3C016347	Chr07: 23806907 .. 23807260 (+)	117	12.89	7.76	51.19	87.61	0.035	Nucleus
*CmNF-YC7*	MELO3C011797	Chr10: 4748948 .. 4753271 (−)	282	31.56	5.20	41.42	63.30	−0.931	Nucleus
*CmNF-YC8*	MELO3C025765	Chr11: 28166692 .. 28169738 (−)	272	30.78	9.52	48.03	56.03	−1.129	Nucleus

**Table 2:** Secondary structure of CmNF-Ys protein.

Proteins	α-Helix %	Extended Strand %	β-Turn %	Random Coil %
CmNF-YA1	18.55	7.55	4.72	69.18
CmNF-YA2	21.74	9.94	3.42	64.90
CmNF-YA3	14.46	16.92	4.62	64.00
CmNF-YA4	19.35	7.04	2.64	70.97
CmNF-YA5	33.14	10.65	6.51	49.70
CmNF-YA6	20.39	16.99	2.91	59.71
CmNF-YB1	46.58	6.21	3.73	43.48
CmNF-YB2	59.15	0.70	3.53	36.62
CmNF-YB3	56.69	6.30	3.94	33.07
CmNF-YB4	59.04	6.67	6.67	27.62
CmNF-YB5	58.12	0.85	5.13	35.90
CmNF-YB6	65.16	6.45	3.23	25.16
CmNF-YB7	47.06	5.23	9.80	37.91
CmNF-YB8	40.00	11.90	4.29	43.81
CmNF-YB9	39.56	3.11	3.56	53.77
CmNF-YB10	51.15	1.72	4.60	42.53
CmNF-YB11	40.00	8.50	6.50	45.00
CmNF-YC1	47.06	5.23	9.80	37.91
CmNF-YC2	47.67	5.02	2.15	45.16
CmNF-YC3	40.79	11.84	5.26	42.11
CmNF-YC4	36.43	8.57	3.57	51.43
CmNF-YC5	46.32	8.46	7.72	37.50
CmNF-YC6	47.01	14.53	5.98	32.48
CmNF-YC7	45.75	1.77	1.77	50.71
CmNF-YC8	33.08	8.27	4.51	54.14

## Data Availability

Not applicable.

## Supplementary Materials

The supporting information can be downloaded at: https://www.mdpi.com/article/10.3390/ijms24086934/s1.
