# Supplementary material for: Genome-Wide Identification and Chilling Stress Analysis of the NF-Y Gene Family in Melon
Source: Int J Mol Sci. 2023 Apr 8;24(8):6934. doi: 10.3390/ijms24086934 (PMC10138816; doi:10.3390/ijms24086934)
Supplement: Supplementary file 1 [file ijms-24-06934-s001.zip › Figures S1 and S2.pdf]

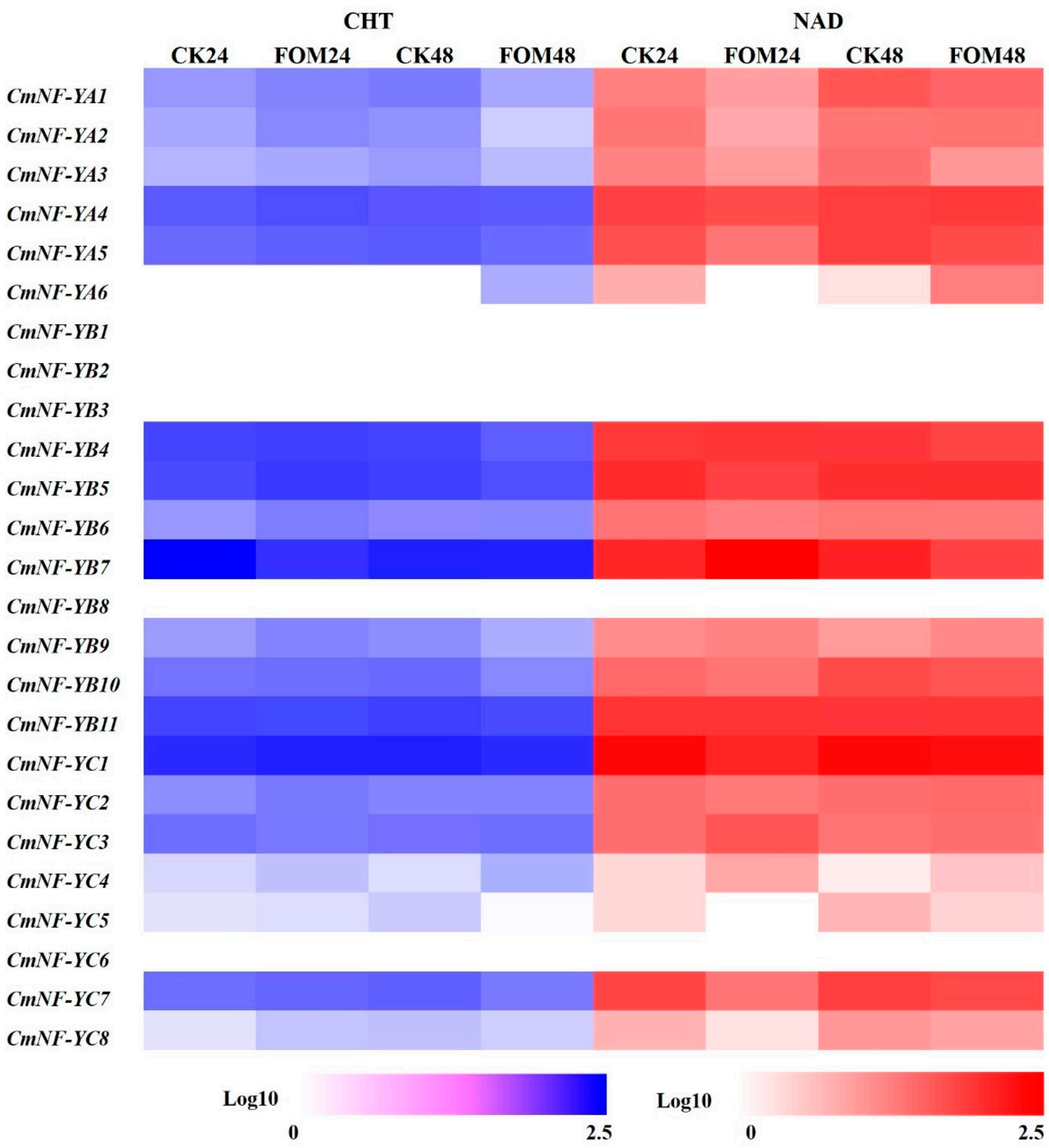

Figure S1. Expression profiles of *CmNF-Ys* after *Fusarium oxysporum* inoculation based on the transcriptome data, the transcriptome data of fusarium wilt tolerance in two genotypes were investigated (PRJEB1551). CHT and NAD were susceptible and resistant to fusarium wilt, respectively. The pathogen of fusarium wilt inoculated was *Fusarium oxysporum* f.sp. melonis Snyd. & Hans race 1.2 (FOM1.2). The raw data were downloaded from the Sequence Read Archive (SRA) database (https://www.ncbi.nlm.nih.gov/sra/).

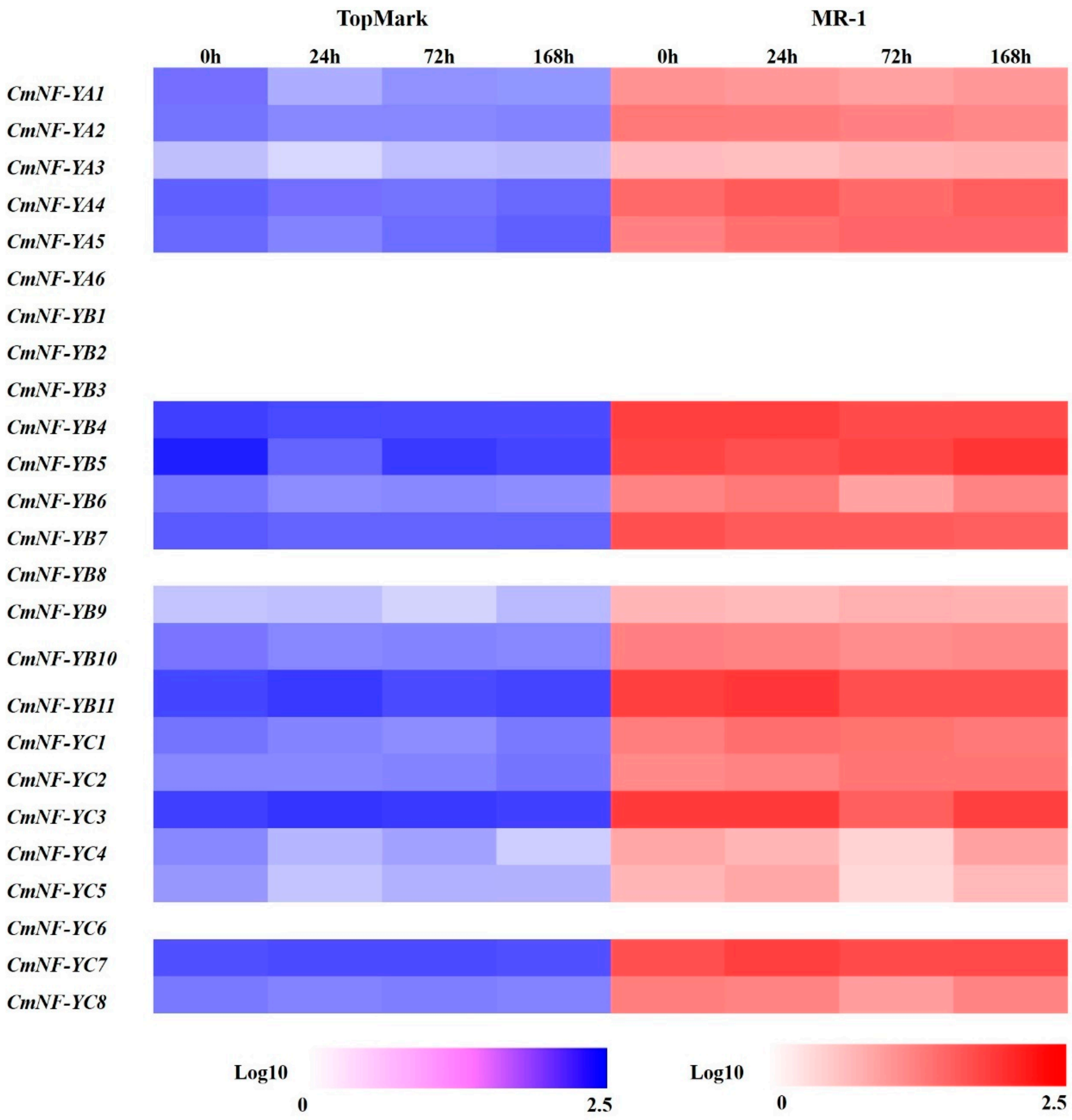

Figure S2. Expression profiles of *CmNF-Ys* under powdery mildew stress based on the transcriptome data, the transcriptome data of PM tolerance in two genotypes were investigated (PRJNA358655). TopMark and MR-1 were susceptible and resistant to powdery mildew, respectively. The raw data were downloaded from the Sequence Read Archive (SRA) database (https://www.ncbi.nlm.nih.gov/sra/).
