# Supplementary material for: Genome-Wide Identification and Chilling Stress Analysis of the NF-Y Gene Family in Melon
Source: Int J Mol Sci. 2023 Apr 8;24(8):6934. doi: 10.3390/ijms24086934 (PMC10138816; doi:10.3390/ijms24086934)
Supplement: Supplementary file 1 [file ijms-24-06934-s001.zip › Table S2. qRT-PCR primers of CmNF-Ys.pdf]

Table S2. qRT-PCR primers of *CmNF-Ys*.

**Gene**	**Primer Sequence 5’-3’**
CmNF-YA1-F	TTCGATCGTCGCCTTCATAATC
CmNF-YA1-R	TGTAGCAAATACTTCGCCCTATC
CmNF-YB6-F	GAAGAAGAACACGCCGAGATAG
CmNF-YB6-R	GTCACGAGAGCACTGAGAAAG
CmNF-YB10-F	TACTAGCGAGGCGAGTGATAAG
CmNF-YB10-R	AAATCCCAACGTGGCCATAG
CmNF-YC1-F	AGGGTTTCTGCTCTGCTTAC
CmNF-YC1-R	CTGAGAACGCTCCGATTGAT
CmNF-YC2-F	GACCTGGAGATACACTTCCTTAC
CmNF-YC2-R	TGGGTCCATAACTGGCTTAC
CmNF-YC5-F	CCAGCAGCTCGGATTAAGAA
CmNF-YC5-R	GTTCCAGTGCCTTTGAGACTA
CmNF-YC7-F	GGGACAACTACCAACCTAACC
CmNF-YC7-R	CTCGACGTGATGTTCCATGT
CmNF-YC8-F	CTCTGTTAGGCCTCGAGTATTG
CmNF-YC8-R	TTAATGCAGGATCCCACCTC
